# An efficiency comparison of different in vitro fertilization methods: IVF, ICSI, and PICSI for embryo development to the blastocyst stage from vitrified porcine immature oocytes

**DOI:** 10.1186/s40813-018-0093-6

**Published:** 2018-08-13

**Authors:** Fahiel Casillas, Miguel Betancourt, Cristina Cuello, Yvonne Ducolomb, Alma López, Lizbeth Juárez-Rojas, Socorro Retana-Márquez

**Affiliations:** 1 Departamento de Biología de la Reproducción, División de Ciencias Biológicas y de la Salud, Universidad Autónoma Metropolitana-Iztapalapa, 09340 CDMX, Mexico; 2 Doctorado en Ciencias Biológicas y de la Salud. Universidad Autónoma Metropolitana-Iztapalapa, 09340 CDMX, Mexico; 3 Departamento de Ciencias de la Salud, División de Ciencias Biológicas y de la Salud, Universidad Autónoma Metropolitana-Iztapalapa, 09340 CDMX, Mexico; 4 Departamento de Medicina y Cirugía Animal, Universidad de Murcia, 30100 Espinardo, Spain

**Keywords:** Porcine, Immature oocytes, Embryo development, Blastocyst, Vitrification, IVF ICSI, PICSI

## Abstract

**Background:**

Most studies carried out to evaluate recovery and development after porcine oocyte vitrification, reported better rates when cryopreserved in embryonic development stages or zygotes, but not in immature oocytes. For this reason, many studies are performed to improve immature oocyte vitrification protocols testing the use of different cryoprotectant concentrations, cooling devices, incubation times; but only a few of them have evaluated which fertilization procedure enhances blastocyst rates in vitrified oocytes. Therefore, this study was aimed to evaluate: 1) if the sperm selection with hyaluronic acid (HA) or polyvinylpyrrolidone (PVP) before injection could play a key role in increasing fertilization and blastocyst formation and 2) the embryo developmental ability and blastocyst production of porcine immature oocytes retrieved after vitrification-warming and co-cultured with granulosa cells during IVM, using different fertilization techniques: in vitro fertilization (IVF), intracytoplasmic sperm injection (ICSI) and conventional ICSI with hyaluronic acid (HA) sperm selection, known as physiological intracytoplasmic sperm injection (PICSI) and.

**Results:**

Sperm selected with HA-PICSI displayed a higher percentage of live/acrosome reacted status compared to those in control and exposed to PVP. Higher dead/acrosome reacted rates were obtained after PVP exposure compared to control and HA. In oocytes, viability significantly decreased after IVM in vitrified oocytes. Besides, IVM rates were not different between control denuded oocytes cultured with granulosa cells (DO-GC) and vitrified oocytes. Regarding fertilization parameters, IVF showed higher percentages of total fertilization rate than those obtained by ICSI and PICSI. However, results demonstrate that PICSI fertilization increased the blastocysts formation rate in control DO-GC and vitrified oocytes compared to IVF and ICSI.

**Conclusions:**

To achieve high blastocyst formation rates from vitrified GV oocytes, it is recommended that sperm should be selected with HA instead of PVP before injection since high viability and acrosome reaction rates were obtained. Also, PICSI fertilization was the best method to produce higher blastocyst rates compared to the IVF and ICSI procedures.

## Background

The improvement of oocyte cryopreservation techniques allows the creation of valuable genetic banks. The development of different oocyte cryopreservation and in vitro fertilization (IVF) methods in swine have significant applications in biomedical research and livestock production [1]. Pigs are considered an important experimental model due to their biological similarities to humans, but also as potential organ donors for xenotransplants [2]. Different cryopreservation methods such as vitrification and slow freezing are applied for organ and tissue preservation; however, gametes represent a major challenge [3]. At present, vitrification is proposed as the best method for oocyte cryopreservation [4], and several studies are performed to improve vitrification protocols testing cryoprotectant agents (CPAs) concentrations [5–7], cooling devices [8–11], co-culture systems [12, 13], incubation times and temperatures [14], but few of them are focused on establishing the best fertilization procedure for vitrified-warmed oocytes [15]. Compared to other meiotic stages and species, porcine oocytes at the germinal vesicle stage (GV) are reported to be more difficult to recover after vitrification due to their high intracytoplasmic lipid content [16, 17]. Low viability, maturation, fertilization and blastocyst rates are obtained after GV oocyte vitrification [10, 18–20], mainly due to different morphological and physiological damage caused by the exposure to high CPAs concentrations, ice crystal formation, abnormal mitochondrial distribution and plasma membrane disruption [21, 22]. Despite this, it has been previously demonstrated that vitrified porcine GV oocytes can complete their nuclear and cytoplasmic maturation, and sustain the subsequent embryo development (ED) until the blastocysts stage. Therefore, the generation of live piglets from vitrified GV oocytes, fertilized with in vitro fertilization (IVF) has already been achieved [10]; however, blastocyst rates were reported to be low (5.2%). In a previous study [15] we used intracytoplasmic sperm injection (ICSI) to fertilize vitrified oocytes because it has the advantage of avoiding polyspermy and zona pellucida (ZP) hardening issues compared to IVF. However, we observed that vitrified oocytes fertilized by conventional IVF displayed higher blastocyst formation rates than those subjected to ICSI. Another study showed that it is possible to obtain blastocysts after ICSI from porcine oocytes vitrified at the GV stage [19]. However, when performing ICSI in control and vitrified oocytes, cleavage and blastocyst rates remain significantly low [19]. This fact could be due to the physiological lacks of ICSI, where some of the sperm checkpoints of natural fertilization are bypassed, such as the sperm-oocyte recognition and acrosome reaction [23–26]. Also, the sperm selection during ICSI is carried out by a visual approach based on motility and sperm morphology, which does not reflect sperm quality. The use of polyvinylpyrrolidone (PVP) is required during ICSI for sperm immobilization; however, this molecule is known to be toxic when injected into the oocyte cytoplasm, possibly affecting fertilization and sperm head decondensation [27]. In this regard, other studies reported low ED rates after ICSI using fresh oocytes [28–30], which are mainly ascribed to an inadequate sperm selection, resulting in sperm chromatin decondensation failures and sex chromosome abnormalities [23, 25]. Therefore, some efforts to increase the efficiency of ICSI are based on the establishment of new sperm selection criteria. For this purpose, some studies performed in fresh oocytes have previously reported the use of ZP [31] or hyaluronic acid (HA) for sperm selection prior to ICSI [32]. The improvement of ICSI using HA is also known as physiological intracytoplasmic sperm injection (PICSI) [33, 34]. One study reported that the use of HA is superior to the use of conventional ICSI in producing chromosomally normal embryos with low aneuploidy rates, suggesting that the ED and quality rates can be significantly improved [35]. However, this study was only performed in fresh oocytes, and to our knowledge, the present study is the first one to evaluate the effect of sperm selection with HA before injection in vitrified porcine immature oocytes. During HA selection, the adhesion of sperm to an HA gel stimulates the natural sperm-granulosa cells recognition and acrosome reaction which improves fertilization [36]. This is highly important because sperm quality is also crucial for the subsequent ED and implantation. Also, other studies reported that sperm bound to HA exhibit increased viability, maturity, acrosome integrity, reduced aneuploidy and DNA fragmentation [37, 38]. For this reason, sperm selection using HA could play a key role by increasing fertilization and ED rates, not only in fresh but also in vitrified immature oocytes. Therefore, the aims of the present study were 1) to evaluate the sperm viability and acrosome status after PVP and HA exposure and 2) to evaluate if the PICSI procedure is a useful tool for improving the fertilization and ED to the blastocyst stage of vitrified porcine GV oocytes compared to IVF and ICSI.

## Methods

### Chemicals culture media and culture conditions

Unless otherwise stated, all reagents were purchased from Sigma Chemical Co. (St. Louis, MO, USA) and different culture media were prepared. For cumulus-oocytes complexes (COCs) collection and washing, Tyrode’s medium containing 10 mM HEPES, 10 mM sodium lactate and 1 mg/mL polyvinyl alcohol (TL-HEPES-PVA) was used [39]. For vitrification and warming, TCM-199-HEPES (product number TCM-199: 170929 ME-044, HEPES: H4034) medium was supplemented with 0.5 mM L-glutamine and 0.1% PVA (V-W medium). To perform in vitro maturation (IVM), the maturation medium (MM) was comprised of TCM-199-Earle’s salts supplemented with 26.2 mM sodium bicarbonate, 0.1% PVA, 3.05 mM D-glucose, 0.91 mM sodium pyruvate, 0.57 mM cysteine and 10 ng/mL EGF (In Vitro, Mexico City). In control denuded oocytes cultured with granulosa cells (control DO-GC) and vitrified oocytes, IVM was performed using an oocyte-granulosa cells co-culture. Granulosa cells were collected from immature oocyte mechanical denudation in MM containing 0.1% hyaluronidase and from the follicular fluid. A total of 1 × 10^6^ granulosa cells were added to each maturation well [40]. Briefly, 500 μL of follicular fluid containing granulosa cells were vortexed in 0.1% hyaluronidase for 5 min, then washed twice in phosphate buffered saline (PBS) and centrifuged at 200 X g for 5 min. The pellet was resuspended in MM and cells were counted on a Neubauer chamber. Finally, 50 μL of the suspension were added to 4-well culture dishes. The medium used for fertilization was modified Tris-buffered (mTBM) containing 3 mM KCl, 13.1 mM NaCl, 7.5 mM CaCl_2_·2H_2_O, 20 mM Tris, 11 mM glucose and 5 mM sodium pyruvate, 0.4% fraction V bovine serum albumin (BSA) and 2.5 mM caffeine [41]. For embryo culture, North Carolina State University-23 (NCSU-23) medium supplemented with 0.4% BSA was used [42]. All culture media and samples were incubated under mineral oil at 38.5 °C with 5% CO_2_ in air and humidity at saturation.

### Oocyte collection

Porcine ovaries were obtained from pre-pubertal Landrace gilts at ¨Los Arcos¨, Edo. de México slaughterhouse and transported to the laboratory in 0.9% NaCl solution at 25 °C. The aforementioned facility has the animal health federal law authorization under the number 6265375. For COCs collection, ovarian follicles between 3 and 6 mm in diameter were aspirated using an 18-gauge needle set to a 10 mL syringe. Oocytes with intact cytoplasm and surrounded by two to four layers of cumulus cells (CC) were selected to perform all experiments.

### Vitrification and warming

For vitrification, immature COCs were denuded mechanically in MM, then washed twice in V-W medium and equilibrated in the first vitrification solution containing 7.5% dimethylsulphoxide (Me_2_SO) and 7.5% ethylene glycol (EG) for 3 min. Later, oocytes were exposed to the second vitrification solution containing 16% Me_2_SO, 16% EG and 0.4 M sucrose for 1 min. Later, at least nine oocytes were immersed in a 2 μL drop and loaded into the Cryolock device (Importadora Mexicana de Materiales para Reproducción Asistida S. A. de C.V. México). Finally, in less than 1 min, the Cryolock was plunged horizontally into liquid nitrogen and the vitrified oocytes were stored for 30 min before warming [12].

For warming, the one-step method was performed [43]. Briefly, the Cryolock was immersed vertically in a four-well dish containing 800 μL of V-W medium with 0.13 M sucrose. Later, warmed oocytes were incubated in the same medium for 5 min and then recovered for IVM [44].

### In vitro maturation (IVM)

Control DO-GC and vitrified-warmed denuded immature oocytes were washed in 500 μL of MM three times. Afterwards, 30 to 40 oocytes were randomly distributed in a four-well dish (Thermo-Scientific Nunc, Rochester NY) containing 500 μL of MM with 0.5 μg/mL LH and 0.5 μg/mL FSH for 44 h and incubated under mineral oil at 38.5 °C with 5% CO_2_ in air and humidity at saturation [42]. Control DO-GC and vitrified oocytes were matured in MM adding a granulosa cell co-culture system as described above.

### Oocyte selection before IVF, ICSI or PICSI

After 44 h of IVM, to perform fertilization, co-cultured granulosa cells were removed in the control DO-GC and vitrification group. Before fertilization, oocytes were evaluated by stereomicroscopy, and only matured oocytes with uniform cytoplasm and intact ZP intended to each experimental group were subjected to IVF, ICSI or PICSI. Oocytes with lysed cytoplasm membranes were considered degenerated and were discarded.

### In vitro fertilization (IVF)

After IVM, mature oocytes were rinsed twice in 500 μL of MM and later in 500 μL of mTBM. Groups of 30 to 40 denuded oocytes from control and vitrified groups were placed into a four-well dish with 50 μL drops of mTBM covered with mineral oil and incubated for 45 min.

To perform insemination, the semen sample was obtained from one Landrace boar, using the gloved hand method at a commercial insemination center, diluted in Duragen (Magapor, México) 1:2 (v:v), then transported to the laboratory at 16 °C within 2 h after collection. Sperm evaluation was performed and motility was determined; only if the semen sample had greater than 80% motile spermatozoa was used. Then, 5 mL of the semen sample were diluted with 5 mL of Dulbecco’s phosphate buffered saline (DPBS; In Vitro, S.A., México) medium supplemented with 0.1% BSA fraction V, 75 μg/mL potassium penicillin G and 50 μg/mL streptomycin sulfate. Then, this suspension was centrifuged (61 X *g* for 5 min). The pellet was discarded and 5 mL of the supernatant were diluted 1:1 (v:v) with DPBS and centrifuged (1900 X *g* for 5 min). The supernatant was discarded, and the pellet was diluted with 10 mL of DPBS and centrifuged twice under the same conditions. Later, the pellet was diluted in 100 μL of mTBM to assess the final sperm concentration (5 X 10^5^ spermatozoa/mL) and after dilution, 50 μL of the suspension were added to the medium containing oocytes. Finally, gametes were co-incubated in mTBM for 6 h.

### Intracytoplasmic sperm injection (ICSI)

Microinjection was carried out using an inverted optical differential interference contrast microscope (Nikon eclipse, TE300, Japan). Holding pipettes (COOK medical, USA) exhibit an external 130 μm and internal 23 μm diameter and injection pipettes (COOK medical, USA) had an outer diameter of 7 μm and an inner diameter of 5.5 μm, both pipettes with an angle of 30°.

For oocyte preparation, an oil covered 35 mm diameter Petri dish (Thermo-Scientific Nunc, Rochester NY) previously incubated at 38.5 °C for 2 h with eight drops of 10 μL mTBM medium for oocytes (3 oocytes/drop) and a drop of 4 μL of mTBM containing 10% PVP (mTBM-PVP) for spermatozoa was used. A 1 μL drop of mTBM medium with the sperm sample was added in the extreme of the 4 μL drop of 10% PVP. To carry out microinjection, progressive motile and normal sperm were immobilized by hitting its tail with the injection needle. Sperm capture was performed by the introduction of the tail into the injection pipette. Then, mature oocytes were aspirated carefully by the holding pipette to prevent polar body damage. The sperm was carefully expelled from inside the injection needle and reloaded for washing to remove the PVP surrounding the sperm before injection. Subsequently, in the position of the three o’clock, the injection pipette was inserted into the oocyte so that the sperm head could be in touch with the cytoplasm to facilitate oocyte activation. To ensure that the injection took place correctly, a small volume of cytoplasm was aspirated and immediately after, the sperm was introduced. Micropipettes were removed and the oocyte was released. Finally, microinjected oocytes were washed twice and IVC [15].

### Physiological intracytoplasmic sperm injection (PICSI)

A 1 μL sperm droplet from the diluted sample described formerly (when performing IVF) was added to the PICSI dish (ORIGIO, Denmark) containing a previously hydrated HA gel drop. To hydrate the gel drop, 1 μL of MM was added in each drop and incubated at 38.5 °C for 3 min. Before injection, the Petri dish containing the sperm sample was incubated at 38.5 °C for 15 min. After that, only spermatozoa bound to the HA drop were selected and subsequently injected into the oocytes as described above by the ICSI method.

### Evaluation of the sperm viability and acrosomal status

Sperm samples used for ICSI or PICSI were stained with propidium iodide (PI) and fluorescein isothiocyanate lectin from the peanut plant, *Arachis hypogaea* (FITC-PNA) for simultaneous evaluation of sperm viability and acrosomal status, respectively. For evaluation, 5 μL of the sperm sample were diluted in 100 μL of mTBM containing 5 μL of PI: 1000 μg/mL solution in distilled water and 5 μL of FITC-PNA: 1000 μg/mL solution in PBS. The sample was homogenized and then incubated for 5 min. Later, 10 μL of the suspension were fixed with 10 μL of 1.6% glutaraldehyde on a slide and evaluated under an epifluorescence microscope (Zeiss Axiostar) with a FITC-TRITC filter set. Sperm observations were classified as follows: live/non-acrosome reacted (A/NAR): positive FITC-PNA at the acrosome and negative PI at the post-acrosomal region. Live/acrosome reacted (A/AR): both FITC-PNA and PI negative. Dead/ non-acrosome reacted (D/NAR): both FITC-PNA and PI positive. Dead/acrosome reacted (D/AR): negative FITC-PNA and positive PI [44].

### Evaluation of oocyte viability

Viability was measured at T 0 h = immediately after collection or vitrification and at T 44 h = after IVM. Oocytes were added in 100 μL drop of 0.5 mg/mL Thiazolyl blue (MTT) diluted in PBS. After 1 h, oocytes were analyzed under a light microscope (Zeiss Axiostar) and classified as viable cells (with purple coloration) and non-viable (colorless).

### Evaluation of maturation and fertilization parameters

**Fig. 1:**
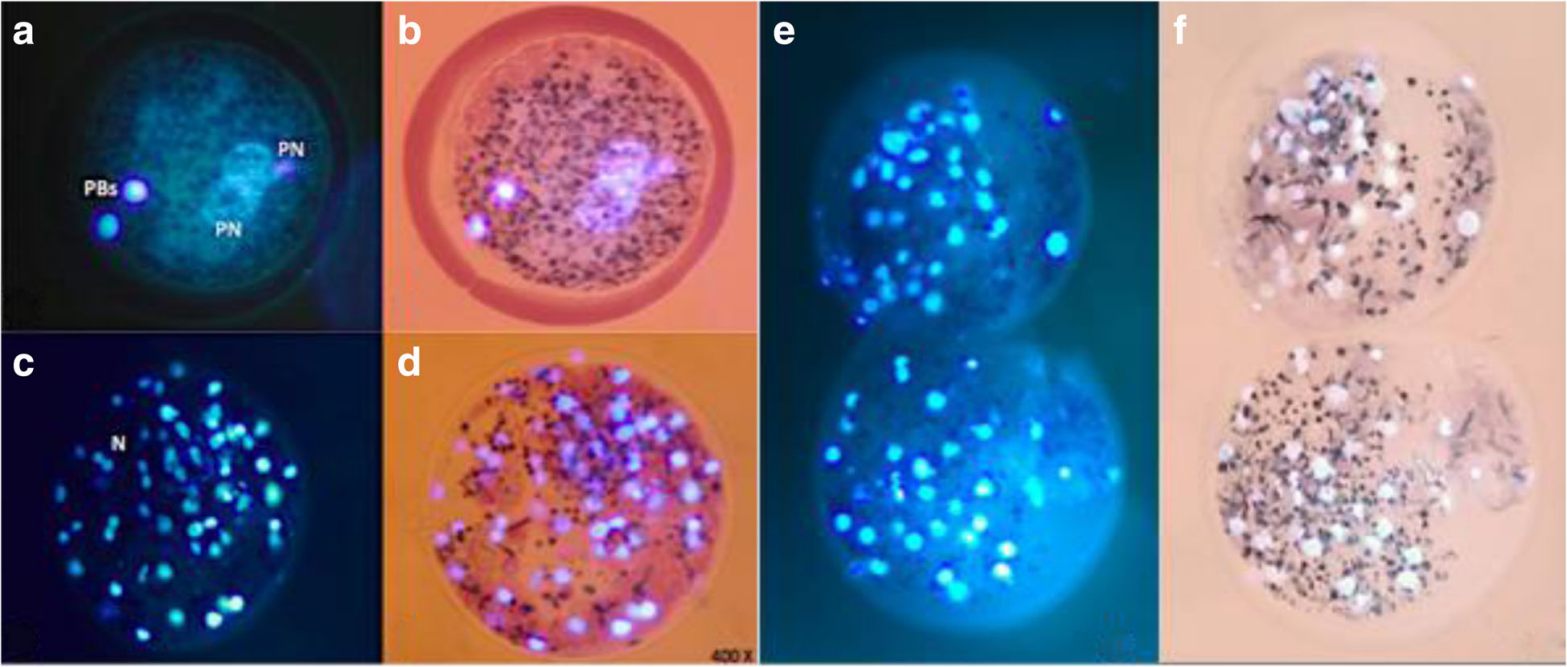
Fertilization and blastocyst assessment by Hoechst-MTT stain. Viable zygote (**a**, **b**), viable blastocyst (**c**, **d**) and dead blastocysts (**e**, **f**). Images were obtained under an epifluorescence microscope with 400X magnification. *PBs* polar bodies, *PN* pronucleus, *N* nucleus

For IVM and IVF parameters, oocytes were stained using 10 μg/mL bisbenzimide (Hoechst 33,342) diluted in PBS for 40 min and washed in PBS. The oocytes were fixed with 2% glutaraldehyde and mounted in a PBS-glycerol solution (1:9). Oocytes and putative zygotes were analyzed under an epifluorescence microscope (Zeiss Axiostar) at 400 X magnification. For maturation parameters evaluation, a random subset of oocytes was fixed after 44 h of IVM and classified as: immature, those oocytes in GV or in metaphase I (MI) and matured, those in metaphase II (MII) [45]. Fertilization parameters were assessed 16 h after IVF or injection in a subset of putative zygotes. Fertilization was evaluated by visualizing pronucleus (PN) formation by the Hoechst staining method. Oocytes were considered activated showing: one pronucleus (PN), monospermic: 2 PN + 2 PBs (Fig. 1, a and b), and polyspermic: > 2PN [46]. Total fertilization rate was calculated as % 2PN + > 2PN oocytes/total oocytes and non-fertilized as % non-pronuclear formation/total oocytes.

### Embryo culture and evaluation of the embryo development, blastocyst quality and viability

**Fig. 2:**
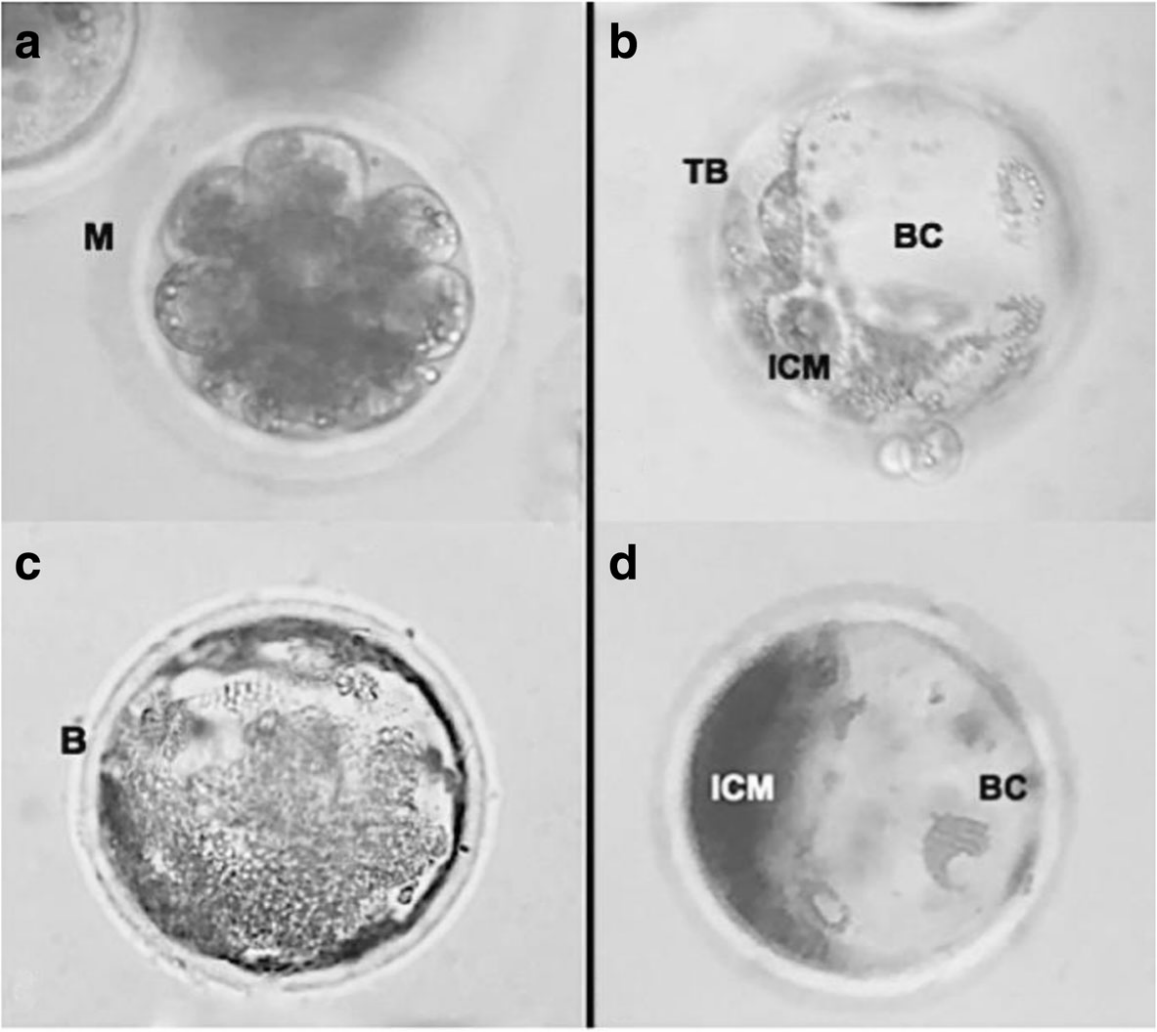
Embryo production. Morulae (**a**) and blastocysts derived from vitrified immature porcine oocytes (**b**–**d**). Images were obtained with an inverted microscope with 400X magnification. *M* morulae, *TB* trophoblast, *B* blastocyst, *BC* blastocyst cavity, *ICM* inner cell mass

After 6 h of gametes co-incubation during IVF or immediately after injection (ICSI and PICSI), 30 to 40 putative zygotes were transferred to four-well dishes containing 500 μL drops of NCSU-23. The embryo cleavage (number of zygotes cleaved per total cultivated) and blastocyst (number of blastocysts per total cultivated) rates were determined at 48 and 168 h after IVC, respectively, by morphological evaluation under an inverted microscope (Olympus Optical) (Fig. 2). At day 7 (d0 = day of IVF or injection) to count the total number of nuclei, blastocysts were stained with 10 μg/mL bisbenzimide (Hoechst 33,342) in MM for 40 min and evaluated (Zeiss Axiostar) at 400 X magnification (Fig. 1, c and e). For cell viability assessment, day 7 blastocysts were added in 100 μL drop of 0.5 mg/mL MTT diluted in PBS. After 1 h, embryos were analyzed under a light microscope (Zeiss Axiostar) and classified as viable (with purple coloration) (Fig. 1, d) and non-viable (colorless) (Fig. 1, f). Results are presented as percentages of viable cells in blastocysts.

### Experimental design

#### Experiment 1: Viability and acrosomal status of PVP exposed spermatozoa and HA-bound sperm

Three replicates were performed to compare the viability and acrosomal status of spermatozoa diluted for ICSI in mTBM and exposed to PVP or HA. The number of sperm evaluated in each replicate was *n* = 200 per treatment. Sperm sample was divided into three groups: 1) non-treated spermatozoa (Control group, *n* = 600), 2) spermatozoa treated with PVP (PVP-ICSI, n = 600) and 3) spermatozoa treated with HA (HA-PICSI, n = 600). Sperm viability and acrosome status were evaluated 15 min after treatment in all groups using the FITC-PNA-PI stain.

#### Experiment 2: Oocyte viability, IVM and IVF parameters obtained after GV oocyte vitrification

Oocytes used to evaluate each parameter correspond to independent samples. At least five experiments were performed to evaluate oocyte viability and maturation in fresh and vitrified oocytes. Viability was assessed immediately after selection (T 0 h) (Control DO-GC, *n* = 80 and Vitrification, *n* = 67) and at the end of IVM (T 44 h) (Control DO-GC, n = 60 and Vitrification, n = 67). After IVM, oocytes were fixed to assess the maturation rate (Control DO-GC, *n* = 100 and Vitrification, *n* = 121). To evaluate IVF parameters, three replicates were performed and the oocytes were distributed in the following groups: 1) non-vitrified oocytes subjected to IVF (Control DO-GC, *n* = 41), ICSI (Control DO-GC, *n* = 30), and PICSI (Control DO-GC, *n* = 45); 2) vitrified GV oocytes matured and subjected to IVF (Vitrification, *n* = 62), ICSI (Vitrification, *n* = 43) and PICSI (Vitrification, *n* = 40). After fertilization, oocytes were cultured for 16 h and fixed to evaluate IVF parameters.

#### Experiment 3: ED and blastocyst quality obtained with vitrified GV oocytes after IVF, ICSI or PICSI

Ten replicates were performed to evaluate ED and three for blastocyst quality. Fresh and vitrified immature oocytes were matured and then randomly allocated in the following groups: 1) non-vitrified oocytes subjected to IVF (Control DO-GC, *n* = 100), ICSI (Control DO-GC, *n* = 74), and PICSI (Control DO-GC, *n* = 60), 2) vitrified GV oocytes subjected to IVF (Vitrification, *n* = 210), ICSI (Vitrification, *n* = 113) and PICSI (Vitrification, *n* = 158). Selected oocytes from each experimental group were subjected to IVF, ICSI or PICSI and cultured as described above to evaluate ED.

### Statistical analysis

To evaluate sperm viability and acrosomal status, oocyte viability, maturation, fertilization parameters, ED and blastocyst quality, data were analysed using ANOVA followed by a non-parametric Duncan test using number cruncher statistical software (NCSS^11^). Percentage data are presented as mean ± standard deviation (SD) values. Differences were considered significant when *P* < 0.05.

## Results

### Spermatozoa viability and acrosomal status

In experiment 1, it was observed that both groups of treated spermatozoa differed (*P* < 0.05) from the control group in terms of the percentage of A/AR spermatozoa (Table 1). Higher A/AR sperm rate was obtained in the HA-PICSI group (*P* < 0.05) when compared to control and PVP. No significant difference was observed between the control and HA-PICSI group in D/AR rates. However, results demonstrate that PVP exposure significantly affects viability and AR (D/AR). The HA-PICSI group displayed lower A/NRA rates than control and PVP (*P* < 0.05) and no differences were obtained in all D/NAR sperm (*P* > 0.05) groups.

**Table 1:** Viability and acrosomal status in spermatozoa selected for ICSI or PICSI

Treatment	Total Spermatozoa n	A/AR (%)	D/AR (%)	A/NAR (%)	D/NAR (%)
Control	600	432 (72 ± 1.4)^a^	29 (5 ± 1.4)^a^	108 (18 ± 1.8)^a^	31 (5 ± 0.4)^a^
PVP-ICSI	600	319 (53 ± 0.9)^b^	180 (30 ± 1.1)^b^	82 (14 ± 2.3)^a^	19 (3 ± 0.2)^a^
HA-PICSI	600	504 (84 ± 2.3)^c^	50 (8 ± 1.9)^a^	42 (7 ± 1.8)^b^	4 (1 ± 0.3)^a^

Percentage data are presented as mean ± SD values

*PVP* Polyvinilpyrrolidone, *HA* Hyaluronic acid, *A/AR* live/acrosome reacted, *D/AR* dead/acrosome reacted, *A/NAR* live/non-acrosome reacted, *D/NAR* dead/non-acrosome reacted, *n* number of sperm examined

^a,b,c^Values in the same column with different letters are significantly different (*P* < 0.05)

### Oocyte viability, IVM and IVF parameters obtained after GV oocyte vitrification

In experiment 2, oocyte viability after vitrification (T 0 h) was not affected compared to control DO-GC; however, it decreased significantly after IVM (T 44 h) up to 66% in the vitrification group (Table 2). Regarding maturation, the percentage of MII oocytes in control DO-GC and vitrified oocytes were not statistically different. However, the percentage of oocytes in GV was higher in control DO-GC compared to vitrified oocytes. Also, GVB (MI + MII) rates were higher (P < 0.05) in vitrified oocytes compared to control DO-GC (Table 3). Regarding fertilization parameters, pronuclear formation was evaluated and 2PN rates were higher in control DO-GC, and vitrified IVF oocytes than ICSI and vitrified PICSI groups. Also, percentages up to 20% of >2PN were obtained after IVF. The total fertilization rate was significantly higher after IVF in control and vitrified oocytes compared to ICSI and PICSI. Lower fertilization rates were obtained after ICSI than IVF and PICSI. Consequently, higher non-fertilized rates (non pronuclear formation) were obtained after ICSI compared to IVF and PICSI procedures (Table 4).

**Table 2:** Viability of porcine oocytes after vitrification and IVM

Treatment	Viability T 0 h (%)	Viability after IVM T 44 h (%)
Control (DO-GC)	75/80 (94 ± 0.8)^a^	52/60 (87 ± 2.3)^a^
Vitrification	65/67 (97 ± 0.2)^a^	44/67 (66 ± 2.1)^b^

Percentage data are presented as mean ± SD values

*DO-GC* Denuded oocytes cultured with granulosa cells

^a,b^Values in the same column with different letters are significantly different (*P* < 0.05)

**Table 3:** IVM of porcine oocytes after vitrification

Treatment	Total Oocyte n	Maturation MII (%)	Meiotic stages (%)		
			GV	MI	GVB
Control (DO-GC)	100	46 (46 ± 4)^a^	49 (49 ± 1.8)^a^	5 (5 ± 0.3)^a^	51 (51 ± 1.2)^a^
Vitrification	121	65 (54 ± 1.2)^a^	44 (36 ± 4.3)^b^	12 (10 ± 0.6)^a^	77 (64 ± 3.2)^b^

Percentage data are presented as mean ± SD values

*DO-GC* Denuded oocytes cultured with granulosa cells, *GV* Germinal vesicle, *MI* Metaphase I, *MII* Metaphase II, *GVB* Germinal vesicle breakdown (MI + MII), *n* number of oocytes examined

^a,b^Values in the same column with different letters are significantly different (P < 0.05)

**Table 4:** In vitro fertilization parameters of fresh and vitrified oocytes

Treatment	Total Oocyte n	Pronuclear formation (%)				
		1PN	2PN + 2 PBs	>2PN	Total Fertilization	Non-fertilized
IVF						
Control (DO-GC)	41	10 (24 ± 1.2)^a^	21 (51 ± 3.4)^a^	8 (20 ± 5)^a^	29 (71 ± 2)^a^	2 (5 ± 1.3)^a^
Vitrification	62	16 (26 ± 3.2)^a^	38 (61 ± 4.3)^a^	8 (13 ± 2.8)^a^	46 (75 ± 3.2)^a^	–
ICSI						
Control (DO-GC)	30	5 (17 ± 8)^a^	10 (33 ± 4.2)^b^	.	10 (33 ± 4.3)^b^	15 (50 ± 1.1)^b^
Vitrification	43	3 (7 ± 1.9)^b^	15 (35 ± 8)^b^	–	15 (35 ± 8)^b^	25 (58 ± 1.2)^b^
PICSI						
Control (DO-GC)	45	10 (22 ± 4)^a^	22 (49 ± 9.9)^a^	–	22 (49 ± 9.9)^c^	13 (29 ± 6.2)^c^
Vitrification	40	8 (20 ± 3)^a^	18 (45 ± 5.6)^c^	–	18 (45 ± 5.6)^c^	14 (35 ± 1.4)^c^

*DO-GC* Denuded oocytes cultured with granulosa cells, *PBs* polar bodies, *PN* pronucleus,

*Total Fertilization =* counted as 2PN + >2PN/total oocytes

*Non-fertilized =* non pronuclear formation/total oocytes

Percentage data are presented as mean ± SD values

^a,b,c^Values in the same column with different letters are significantly different (P < 0.05)

### Blastocyst formation obtained with vitrified GV oocytes

In experiment 3, vitrification did not impair the embryo cleavage rates obtained after IVF, ICSI and PICSI (Table 5). Cleavage rates were not statistically different between IVF and PICSI procedures. However, cleavage decreased (*P* < 0.05) after ICSI. Blastocyst formation was significantly higher after PICSI compared to IVF and ICSI. Also, higher (P < 0.05) percentages of viable cells in blastocysts were obtained after PICSI and ICSI control DO-GC group compared to IVF. In terms of total number of nuclei, IVF and PICSI were not statistically different; however, it decreased significantly after ICSI.

**Table 5:** In vitro embryo development and blastocyst quality of fresh and vitrified oocytes

Treatment	Total n	Cleavage (%)	Blastocyst (%)	Viable cells in blastocysts %	Total no. of nuclei (means ± S.D.)
IVF					
Control (DO-GC)	100	73 (73 ± 3.4)^a^	15 (15 ± 1.2)^a^	82 ± 3^a^	50 ± 0.6^a^
Vitrification	210	142 (68 ± 2)^a^	30 (14 ± 1.8)^a^	70 ± 5^b^	46 ± 3^a^
ICSI					
Control (DO-GC)	74	33 (45 ± 2)^b^	9 (12 ± 0.3)^a^	100^c^	44 ± 0.9^b^
Vitrification	113	45 (40 ± 9)^b^	10 (9 ± 0.2)^b^	42 ± 5^d^	41 ± 2^b^
PICSI					
Control (DO-GC)	60	38 (63 ± 2)^a^	18 (30 ± 1.5)^c^	100^c^	50 ± 2.5^a^
Vitrification	158	99 (63 ± 3.5)^a^	39 (25 ± 3)^c^	100^c^	54 ± 5^a^

*DO-GC* Denuded oocytes cultured with granulosa cells, *n* number of embryos examined

Cell viability was considered as the percentage of viable cells in blastocysts

Percentage data are presented as mean ± SD values

Cleavage = number of zygotes cleaved per total cultivated

Blastocyst = number of blastocysts per total cultivated

^a,b,c,d^Values in the same column with different letters are significantly different (P < 0.05)

## Discussion

Experiment 1 results indicated that HA-PICSI sperm displayed higher A/AR rates than control and PVP exposed sperm. For fertilization, high A/AR rates are needed to promote sperm head decondensation. Also, reduced D/AR, A/NAR and D/NAR sperm were obtained after HA exposure. Compared to IVF and PICSI, during ICSI, PVP is often used for sperm manipulation decreasing motility and facilitating capture. However, it was reported that PVP could be toxic for the spermatozoa [47], reducing fertilization [27], male PN formation and blastocyst development [48]. These observations agree with our results, where the PVP exposed spermatozoa displayed a higher proportion of D/AR sperm than those exposed to HA and control, suggesting that the HA does not affect sperm viability. Also, the HA-binding mechanism is related to sperm maturity [49]. Only mature sperm have HA specific ligand-receptors, which are implicated in the fertilization potential. Thus, conventional sperm preparation techniques prior to fertilization [50] such as removal of seminal plasma (sperm washing), filtration, centrifugation, swim up, PVP, and observational selection based on motility, have important limitations. These procedures do not select functional, mature and competent spermatozoa, and are possibly involved in reducing sperm viability, fertilization and ED [38, 51]. Important aspects of sperm functions such as motility; maturation and capacitation appear to be partially mediated through HA [52]. In human and porcine oviduct fluid, HA is also found [36], suggesting that great amounts of HA are in contact with the sperm through the oviduct, possibly maintaining their viability until fertilization. Interactions between the oviduct fluid and sperm are required for fertilization. Only mature sperm have hyaluronic specific ligand-receptor that facilitates HA-binding, hyaluronidase activity, ZP recognition and acrosome reaction [53, 54]. Therefore, our results demonstrate that sperm viability is less affected when sperm are exposed to HA (*P* < 0.05).

Experiment 2 results demonstrate that oocyte viability is not affected immediately after vitrification (T 0 h). However, it was significantly reduced after IVM (T 44 h) in the vitrification group compared to control DO-GC. In agreement, other studies reported that viability decreases after IVM in vitrified porcine [55] and goat oocytes and embryos [56]. This fact could be due to the high sensitivity of the GV oocytes during vitrification compared to other meiotic or developmental stages [22], mainly because of their high lipid content [16], reducing CPAs permeation, causing cell damage and lowering viability [19]. Other studies support that the oocyte viability after vitrification decreases after IVC [12, 15, 57, 58]. This fact could be since, during IVC, O_2_ and reactive oxygen species levels increase, affecting oocyte viability [59]. Also, buffalo [60] and porcine oocytes [61] exhibit an increased intracytoplasmic lipid content [16], affecting in vitrified oocytes the glutathione levels and increased production of H_2_O_2_, decreasing viability rates [61]. Concerning maturation, in the present study, it was possible to obtain maturation rates up to 46% in control DO-GC and 54% after vitrification. In agreement, several studies reported rates from 3 to 61% MII oocytes after GV oocyte vitrification [10, 18, 19, 58]. Also, our results indicate that maturation was not affected in vitrified GV oocytes compared to control DO-GC. Other studies indicate that porcine GV oocytes are less cryotolerant. Other factors responsible for decreasing IVM rates after vitrification include ZP damage, reduced mitochondrial matrix density and irreversible cytoskeleton damage [22, 62]. In the present study, the DO co-cultured with isolated CC can partially recover meiotic and developmental competence. These cells have a pH regulatory mechanism during culture and paracrine factors display antioxidant properties allowing maturation [63, 64]. However, co-culture of DO with granulosa cells does not reestablish gap junctions and IVM rates remain low. Premature nuclear maturation, oocyte aging and GAP junction damage occurs after vitrification [18, 22, 65]. Regarding fertilization, several attempts were made to increase IVF and ED rates of vitrified porcine GV oocytes [10, 12, 15]. However, embryo production rates remain low due to a high incidence of vitrification-warming injuries [22]. In the present study, higher 1PN + 2PBs rates were obtained after IVF, control ICSI and PICSI than the vitrified ICSI group, suggesting that low oocyte activation and insufficient sperm head decondensation is obtained after ICSI. When evaluating pronuclear formation, higher 2PN rates were obtained after IVF and PICSI than the ICSI procedure. Other studies performed in control and vitrified GV oocytes reported 2PN rates up to 43 and 33%, respectively [58], 23 and 13%, respectively [66] and fresh oocytes [67, 68]. Nevertheless, significantly reduced male PN formation was obtained after ICSI. Also, results indicate that the IVF procedure increases total fertilization rates compared to ICSI and PICSI. However, with the IVF procedure, polyspermy (>2PN) rates up to 13–20% are obtained and polyspermic fertilization is one of the leading causes in producing low quality embryos. Therefore, the IVF method selection, sperm and oocyte factors could have detrimental effects on the fertilization and ED potential. In agreement to our results, another study reported that reduced oocyte activation and male PN formation are the main causes of low ICSI efficiency [69]. Results obtained in the present study demonstrate that less PN formation (non-fertilized oocytes) is obtained after ICSI compared to IVF and PICSI. Therefore, sperm selection with PVP may lead to a reduced PN formation. The ICSI efficiency was previously tested; results obtained in other studies showed 55% of 2PN fertilization in control oocytes that can be improved using roscovitine up to 72% [2]. Also, oocyte activation with ionomycin in parthenogenetic oocytes or calcium ionophore and sperm selection before injection with ZP binding were performed in porcine [70, 71] and ovine oocytes [44]. However, significantly low fertilization and cleavage rates were obtained. In the present study, the use of HA increased 2PN formation up to 45%. Recently, it was reported that the use of lipase to select and inject oocytes, resulted in 29% of 2PN formation [48]. In addition, this study also evaluated the efficiency of PVP, reporting 24% of 2PN formation [48], similar to those obtained in the present study (35%). Fertilization and ED success depends on the quality of the spermatozoa selected for injection, but this is not possible to achieve by ICSI.

In experiment 3, regarding cleavage results, IVF and PICSI were not statistically different. However, significantly reduced cleavage rates were obtained after ICSI. Therefore, results demonstrate that sperm selection is crucial for improving ED. PVP sperm selection reduces viability, and could increase DNA fragmentation rates resulting in less oocyte activation, PN formation and ED. In agreement, other studies reported low blastocyst production after ICSI, in fresh oocytes (4–14%) [2, 19]. Our results demonstrate that cleavage and blastocyst rates are significantly affected after ICSI compared to IVF and PICSI. In contrast, another study reported blastocyst rates up to 40% in pigs after ICSI; however, different culture media and BSA supplementation were used [72]. When performing PICSI, blastocyst formation was improved in control DO-GC (30%) and in vitrified oocytes (25%). To increase ED rates, other studies add 10% of fetal bovine serum (FBS) or porcine follicular fluid (pFF) during IVM or IVF culture [35, 58, 73]; however, we avoided its use. In the present study, we obtained similar results in terms of cleavage rate with fresh oocytes in all treatments without using FBS or pFF. Supplementation with FBS or pFF has important limitations since pathogens such as viruses are present. Regarding blastocyst viability and quality, results indicate that viable day 7 blastocysts can be obtained by PICSI of matured oocytes derived from vitrified GV oocytes. Higher percentages of viable cells in blastocysts and a total number of nuclei were obtained by PICSI than when using IVF and ICSI. Our results demonstrate that sperm selection with HA, ensures the injection of a sperm that has completed its maturation and that is able to recognize and attach to the HA, improving blastocysts formation. However, sperm selection with PVP during ICSI can significantly reduce the oocyte developmental potential to the blastocyst stage. Therefore, superior quality blastocysts can be obtained after HA sperm selection during PICSI. It has been stated that the contribution of sperm towards embryogenesis can be divided into two periods: the early period (fertilization to < 8-cell stage) and the late period (8-cell stage to birth) [74]. In the early period, an inadequate sperm selection can affect fertilization, syngamy and the mitotic division [75, 76]. In the late period, sperm can influence embryogenesis by a genome way. If the genome is altered by DNA fragmentation, it may result in a poor blastocyst development, lower implantation, early pregnancy loss or abortion [74]. According to this, other studies with PICSI fertilization have reported that HA-bound injected sperm increased embryo production in human [34]. But also, low pregnancy loss, high pregnancy rates [77, 78], and high DNA integrity [79] are obtained by PICSI. Therefore, our results demonstrate that sperm selection with HA improves blastocysts rates not only in control but also in vitrified oocytes. Compared to PVP-ICSI, sperm selection with HA improved blastocyst formation. The PICSI mechanism by which ED is improved is that only mature and competent sperm hold hyaluronan specific ligand receptors, facilitating fertilization. HA-selected sperm exhibit normal head morphology, reduced DNA fragmentation, reduced chromosomal aneuploidy rates and better fertilization potential [37]. In contrast, PVP has no selective function and its use can cause DNA fragmentation rates up to 11% [34] compared to 5.3% with HA. Also, higher percentages of spermatozoa with normal nucleus are selected with HA compared to PVP (14.5% vs. 11%, respectively) [34]. Consequently, oocyte fertilization with arrested sperm maturity and DNA damage may lead to a reduced blastocyst development. Also, lower oocyte activation and PN formation rates were obtained after ICSI compared to PICSI, reducing the ED potential. Therefore, the present study suggests that the PICSI procedure is the best method to fertilize and produce blastocysts from vitrified GV oocytes. This procedure compared to the conventional IVF in porcine oocytes has several advantages: 1) during PICSI, polyspermy is avoided, 2) only one selected sperm is used per oocyte and 3) sperm selection allows the injection of a high-quality sperm. The IVP of porcine blastocysts has been difficult to achieve mainly in vitrified GV oocytes. However, if nuclear maturation is performed with a granulosa cell co-culture system and sperm are selected with HA before injection, blastocyst production can be improved. The advantages of cryopreserving GV oocytes compared to other meiotic or developmental stages are that they can be collected in a greater quantity than MII oocytes, allowing the production of a high number of blastocysts for embryo transfer. Also, to obtain GV oocytes, no ovarian stimulation is required. They can be obtained from prepubescent females; however, IVM is required.

## Conclusions

To achieve high blastocyst formation rates from vitrified GV oocytes, it is recommended that sperm should be selected with HA instead of PVP before injection since high viability and acrosome reaction rates were obtained. Also, PICSI fertilization was the best method to produce higher blastocyst rates compared to the IVF and ICSI procedures.

## Abbreviations

A/AR: Live/acrosome reacted
A/NAR: Live/non-acrosome reacted
ART: Assisted reproduction technologies
BC: Blastocyst cavity
BSA: Bovine serum albumin
CC: Cumulus cells
COCs: Cumulus-oocyte complexes
CPAs: Cryoprotectant agents
D/AR: Dead/acrosome reacted
D/NAR: Dead/ non-acrosome reacted
DO: Denuded oocytes
DO-GC: Denuded oocytes cultured with granulosa cells
DPBS: Dulbecco’s phosphate buffered saline
ED: Embryo development
EG: Ethylene glycol
EGF: Epidermal growth factor
FBS: Fetal bovine serum
FITC-PNA: Fluorescein isothiocyanate-peanut agglutinin
FSH: Follicle stimulating hormone
GAGs: Glycosaminoglycan’s
GV: Germinal vesicle
GVB: Germinal vesicle breakdown
HA: Hyaluronic acid
ICM: Inner cell mass
ICSI: Intracytoplasmic sperm injection
IVC: In vitro culture
IVF: In vitro fertilization
IVM: In vitro maturation
LH: Luteinizing hormone
M: Morulae
Me_2_SO: Dimethylsulphoxide
MI: Metaphase I
MII: Metaphase II
MM: Maturation medium
mTBM: Tris-buffered medium
MTT: Thiazolyl blue
N: Nucleus
NCSU-23: North Carolina State University 23
PBS: Phosphate buffered saline
PBs: Polar bodies
pFF: Porcine follicular fluid
PI: Propidium iodide
PICSI: Physiological intracytoplasmic sperm injection
PN: Pronucleus
PVA: Polyvinyl alcohol
PVP: Polyvinylpyrrolidone
SD: Standard deviation
TB: Trophoblast
V-W: Vitrification and warming
ZP: Zona pellucida
